# Using citizen science data to inform the relative sensitivity of waterbirds to natural versus human‐dominated landscapes in China

**DOI:** 10.1002/ece3.6449

**Published:** 2020-06-09

**Authors:** Houlang Duan, Shaoxia Xia, Xiubo Yu, Yu Liu, Jiakun Teng, Yuehan Dou

**Affiliations:** ^1^ Key Laboratory of Ecosystem Network Observation and Modeling Institute of Geographic Sciences and Natural Resources Research Chinese Academy of Sciences Beijing China; ^2^ College of Resources and Environment University of Chinese Academy of Sciences Beijing China; ^3^ Land Use Planning Group Wageningen University and Research Wageningen The Netherlands

**Keywords:** China, functional group, human‐dominated landscapes, natural landscapes, sensitivity, waterbirds

## Abstract

Habitat loss is widely regarded as one of the most destructive factors threatening native biodiversity. Because migratory waterbirds include some of the most globally endangered species, information on their sensitivity to landscape would benefit their conservation. While citizen science data on waterbird species occurrence are subjected to various biases, their appropriate interpretation can provide information of benefit to species conservation. We apply a bootstrapping procedure to citizen science data to reduce sampling biases and report the relative sensitivity of waterbird species to natural versus human‐dominated landscapes. Analyses are performed on 30,491 data records for 69 waterbird species referred to five functional groups observed in China between 2000 and 2018. Of these taxa, 30 species (43.5%) are significantly associated with natural landscapes, more so for cranes, geese, and ducks than for shorebirds and herons. The relationship between land association and the threat status of waterbirds is significant when the range size of species is considered as the mediator, and the higher the land association, the higher the threat status. Sensitive species significantly associated with natural landscapes are eight times more likely to be classified as National Protected Species (NPS) Classes I or II than less sensitive species significantly associated with human‐dominated landscapes. We demonstrate the potential for citizen science data to assist in conservation planning in the context of landscape changes. Our methods might assist others to obtain information to help relieve species decline and extinction.

## Graphical abstract

We apply a bootstrapping procedure to citizen science data to reduce sampling bias and report on the relative sensitivity of waterbirds species to natural versus human‐dominated landscapes in China.

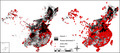

## 1 INTRODUCTION

Global biodiversity loss is of worldwide scientific concern (Johnson et al., 2017). Land cover changes caused by human activities represent one of the greatest threats to biodiversity (Pimm et al., 2014). In extreme cases, habitat loss may cause species extinction (Brooks et al., 2010; Lehosmaa et al., 2017). However, habitat loss affects species differently (Lei et al., 2019; Naujokaitis‐Lewis, Curtis, Arcese, & Rosenfeld, 2010), with some species more sensitive to landscapes than others, due to ecological, physiological, or behavioral traits (Callaghan et al., 2019). Populations of more sensitive species are often at higher risk of decline as a consequence of human activities (Todd, Rose, Price, & Dorcas, 2016).

Waterbirds are a significant component of global biodiversity. The East Asian–Australasian Flyway (EAAF) provides habitat to over 50 million migratory waterbirds (EAAF, 2017). China's wetlands are key habitats in this EAAF, yet populations of some species dependent on this region have declined sharply, due to accelerated, recent losses in natural habitats (Si et al., 2018; Studds et al., 2017; Yang, Ma, Thompson, & Flower, 2017), although other factors may also have contributed.

An understanding of the sensitivities of waterbird taxa to landscapes would prove valuable for conservation. Such information could be used to prioritize taxa that are particularly at risk for conservation and to enact taxon‐specific conservation efforts. Unfortunately, detailed information on the vulnerability or sensitivity of waterbird taxa to disturbance is hampered by data deficiency, especially the lack of any large spatial scale long‐term systematic surveys (Johnson & Gillingham, 2008; Todd, Nowakowski, Rose, & Price, 2017). This is where citizen science data might fill the gaps.

Citizen science data are routinely collected over large spatial and temporal scales and have been widely used to inform biodiversity protection (Crall et al., 2011; Soroye, Ahmed, & Kerr, 2018; Xu et al., 2019). Such data can include the likes of, but not be limited to, species records (names), location data (longitude, latitude, and place name), abundance records, behavioral notes, and survey dates. Using these data directly presents challenges for analysis (Devictor, Whittaker, & Beltrame, 2010; Dickinson, Zuckerberg, & Bonter, 2010), particularly because nonsystematic survey methods are often used. Solutions to this problem would enable improved use of these data in conservation and biodiversity assessments (Broms, Johnson, Altwegg, & Conquest, 2014; Higa et al., 2015).

Randomization procedures, which generate comparisons of species occurrence with subsamples of all occurrences in their functional groups, represent one of a number of nonparametric statistical techniques commonly known as resampling methods. Randomization has been used frequently to address the problem of sampling bias in citizen science data, improving the accuracy of interpretations based on such data (Chernick & LaBudde, 2011; Weisshaupt & Rodríguez‐Pérez, 2017).

In the study, we aim to investigate the relative sensitivity of waterbird taxa to landscapes, using citizen science data. We hypothesize that sensitive species that are threatened with extinction will have distributions primarily associated with natural habitats. In contrast, species with distributions primarily associated with human‐dominated areas (or those with no significant land association), that are presumably less sensitive to human activities, will be less likely to be threatened. We focus on 69 waterbird species, of which 14 are considered threatened. We grouped these 69 species to one of five functional groups based on their feeding habits (Cumming, Paxton, King, & Beuster, 2012; Del Hoyo, Elliot, & Christie, 1992). The extent to which each species within a functional group is associated with a habitat type is described based on whether a taxon is: significantly associated with natural landscape, significantly associated with human‐dominated landscape, or whether it shows no significant association with any land‐type use. We evaluate the relationship between land association and threat status of species and also test whether more species that are significantly associated with natural landscapes can be protected by legislation of wildlife conservation.

## 2 METHODS

### 2.1 Citizen science data

Waterbird occurrence records in China between 2000 and 2018 were sourced from eBird (https://ebird.org/home), the Global Biodiversity Information Network (GBIF) (http://www.gbifchina.org/), and BirdReport (http://www.birdreport.cn/). Downloaded records include species names, longitudes, latitudes, place names, and survey dates. These online data records have been typically checked and filtered by ornithological enthusiasts and website administrators, so errors, such as mistakes in identification, are uncommon (Chandler et al., 2017; Li, Liang, Gong, Liu, & Liang, 2013; Sullivan et al., 2014). Observations prior to time of 2000 are excluded, as our land use and land cover data (2.2.1) do not cover this period.

To address spatial and temporal biases, raw data were vetted before use. We first manually verified coordinates that deviated significantly from an actual place name using Google Maps 6.5 (https://www.google.com/maps), using the center of the place name as the site coordinate. To assess accuracy, we randomly selected and input 30% of our sites into Google Earth 6.0 for visual verification and determined site accuracy to exceed 90%. Bootstrapping can reduce biases introduced by more data being recorded from popular locations or from people being more likely to report observations of one species than another. We also checked for temporal bias. It was unusual to find a high number of occurrences of a species within a certain time period. We removed duplicate records from sites with the same longitude and latitude from our dataset. Finally, we excluded any species with fewer than 20 observations.

Our cleansed dataset contained 30,491 records for 69 species, comprising: (a) 28 shorebirds (invertebrate eaters, mainly inhabiting muddy areas and shallow water); (b) 4 cranes (tuber eaters, mainly inhabiting shallow water and wet mudflats); (c) 5 geese (short grass foragers, mainly inhabiting wet meadows); (d) 23 ducks (seed and aquatic vegetation eaters, mainly inhabiting shallow water); and (e) 9 herons (fish eaters) (Kear, 2005; Ma, Cai, Li, & Chen, 2010; Wang et al., 2013). Three of 69 species were classified as critically endangered (CR), five as endangered (EN), six as vulnerable (VU), nine as near threatened (NT), and 46 as of least concern (LC) (IUCN, 2019) (Appendix S1).

### 2.2 Statistical analysis

#### 2.2.1 Natural landscape metric

We use the 2000 National Terrestrial Ecosystem Spatial Distribution Data set to create a natural landscape (NL) metric, which indicates the degree of landscape naturalness. This dataset was mainly obtained by visual interpretation of 873 scenes (resolution 30 m × 30 m) from Landsat Thematic Mapper/Enhanced Thematic Mapper. Land types can be divided into six first‐level classes according to the utilization property of land resources and 24 s‐level classes according to the natural property of land resources, http://www.resdc.cn/Default.aspx.

First, for each 30 m × 30 m cell, we classified “natural landscapes,” represented by forest, grassland, wetland, lake, and canals and gave it a raster value of 1; seminatural landscapes, represented by farmland, saltpans, and that used for aquaculture, were given a raster value of 0.5; human‐dominated landscapes, represented by urban areas or built‐up land and roads, were given a raster value of 0.

Second, we defined corresponding *I_j_* as the proportion of natural land cover at each raster cell location and using an inverse distance‐weighted nearest‐neighbor approach by Equation (1). The proportion of natural land cover at a raster cell location can be regarded as the probability that it is natural.

(1) $$I_{j}=\sum_{i}^{n}P_{i}P_{c}/n$$

**FIGURE 1:**
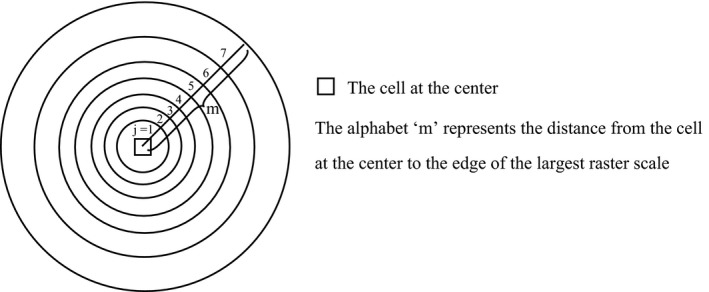
Calculating natural landscape values by calculating the proportion of natural cover at seven raster scales using neighborhoods of 0.27 km (*j* = 1), 0.76 km (*j* = 2), 2.43 km (*j* = 3), 7.19 km (*j* = 4), 21.86 km (*j* = 5), 64.70 km (*j* = 6), and 197.41 km (*j* = 7)

The *I_j_* represents the proportion of natural land cover at a raster location; the *P_c_* and *P_i_* are the cell values in the center and neighboring cells, respectively, at raster scale *j*. For this study, *j* = 1 is set to 0.27 km, so the central cell is surrounded by 81 neighborhood cells (*n* = 81). The other six scales are 0.76 km, 2.43 km, 7.19 km, 21.86 km, 64.70 km, and 197.41 km (Riitters et al., 2002) (Figure 1). The operation will stop when the distance from the cell at the center to the edge of the largest raster scale reaches 109 km (the alphabet “m” in the Figure 1) (Theobald, 2010).

Finally, we used the average *I_j_* of seven scales as the natural landscape value (Equation 2). Using this classification scheme, at each raster scale, if a cell at the center is composed entirely of natural landscape, with all adjacent cells also natural landscape, it receives an NL value of 1; if a cell at the center is composed entirely of human‐dominated landscape, with all adjacent cells also human‐dominated landscape, it receives an NL value of 0.

(2) $$\text{NL}=\sum_{j=1}^{k}I_{j}/k$$

NL represents the natural landscape value, and the *k* is the number of raster scale, *k* = 7.

#### 2.2.2 Bootstrapping procedure

A bootstrapping approach was used to minimize sample bias and clarify the relative sensitivity of waterbirds to natural versus human‐dominated landscapes (Phillips et al., 2009). This method reduces possible sampling bias by comparing the mean NL value of targeted species' occurrence records with the 1,000 averages of randomly selected 1,000 background samples in the buffer area of targeted species. For example, if occurrence data for a species are extracted only from easily surveyed regions, then background data should be extracted from these same regions also (Todd et al., 2016, 2017).

We assumed that species within each functional group would be more likely found with similar search effort and have a similar likelihood of being reported. Using the occurrence records of species in the same functional group as background data can decrease sample bias. For targeted species, we first extracted the NL value for each occurrence of it according to the real distribution in each cell. Then, for background data, we considered all occurrence records of the target species as the centers of the circles and selected the 20 km (a distance that covers most occurrence records of species in the same functional group, according to the real distributions of species studied) as the radius of each circle to create buffers. We then extracted all NL values for unduplicated occurrences (i.e., occurrences at different longitudes and latitudes) of all species in the same functional group within the buffers as the background data for the targeted species. We randomly selected 1,000 samples of these NL values: The number of unduplicated occurrences of each sample was the same as the number of occurrences of targeted species.

**FIGURE 2:**
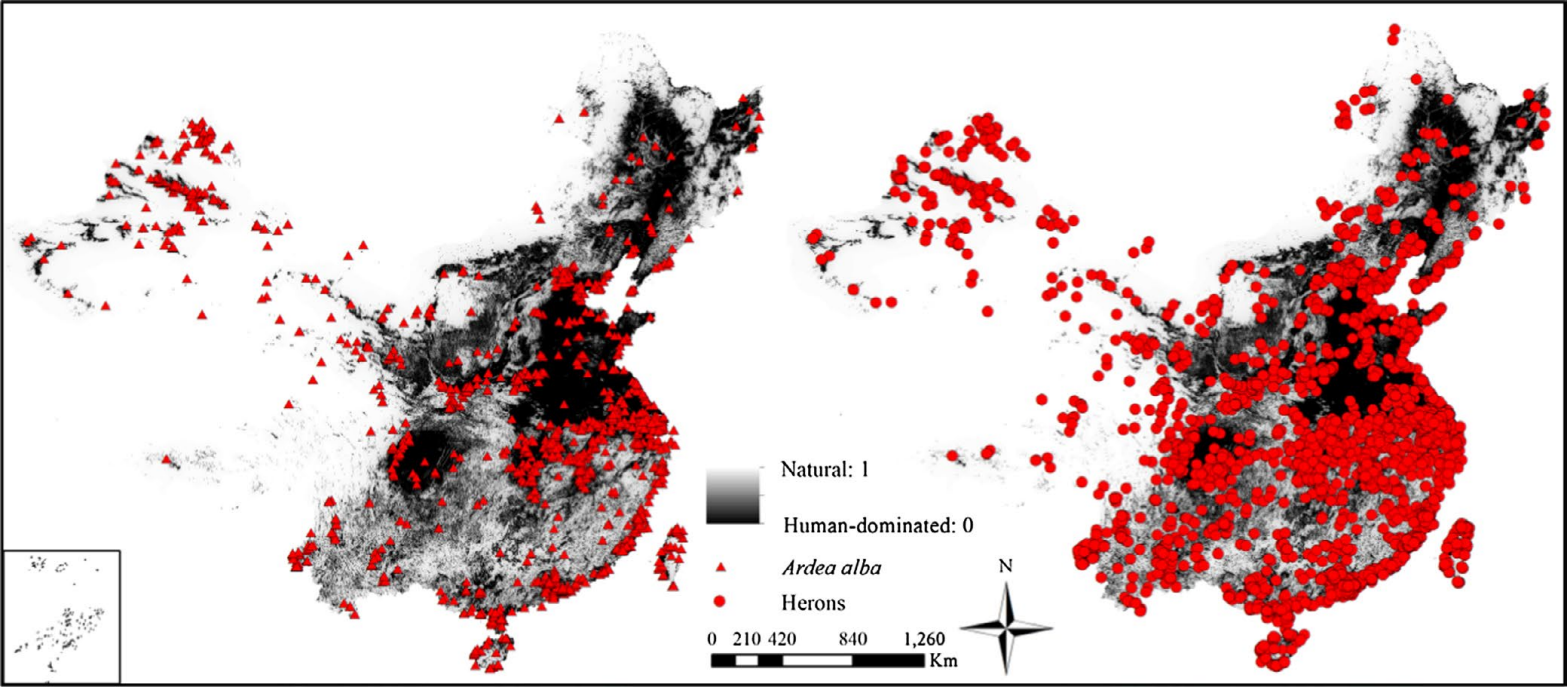
Natural landscape metric layer for China. Occurrences of only *Ardea alba* (left); all heron occurrences (right)

We calculated the mean NL value of each sample and acquired 1,000 averages and then ordered them from lowest to highest. We then compared the real mean NL value of targeted species with the 0.025 and 0.975 quantiles of the 1,000 averages and assigned species found in the lower or upper 2.5% of the distribution as significantly associated with natural or with human‐dominated landscapes, respectively. Otherwise, there was no significant association with landscape type. For example, for 1,603 unduplicated occurrences of the heron *Ardea alba* (Figure 2, left image), we first calculated the mean NL value based on the 1,603 occurrence records of targeted species. Then, we considered 1,603 occurrence records of *A. alba* as the centers of the circles and selected the 20 km as the radius of each circle to create buffers. Finally extracted the NL value of all unduplicated occurrences of all species of herons located within buffers (Figure 2, right image), of which there were 3,530 records. We randomly selected 1,603 occurrence records from 3,530 occurrence records and repeated this 1,000 times. For each time, we calculated the mean NL of 1,603 occurrence records, and acquired 1,000 averages and then ordered the 1,000 averages from lowest to highest. Finally, we compared the mean NL value for *A. alba* with the 975th or 25th of the 1,000 averages, according to the bootstrapping procedure.

A standardized effect size (SES) was calculated as a measure of the extent to which the actual mean NL value of a target species was above or below the mean NL value of the 1,000 averages, based on the bootstrapping iteration (Cooke, Bates, & Eigenbrod, 2019). A higher SES indicates a higher association with natural landscapes. The specific function is:

(3) $$\text{SES}=\frac{\bar{{\text{NL}}_{\text{actual}}}-\bar{{\text{NL}}_{1000}}}{\bar{{\text{SD}}_{1000}}}$$

where $\bar{{\text{NL}}_{\text{actual}}}$ represents the actual mean NL of a target species, and $\bar{{\text{NL}}_{1000}}$ and $\bar{{\text{SD}}_{1000}}$ represent the mean NL value and standard deviation of the 1,000 averages, respectively.

We performed bootstrapping procedures at the functional group level in a manner similar to that we performed at the species level. For example, we used all occurrences of four species of cranes to calculate a mean NL for the presence of this functional group. Then, considered all occurrence records of four species of cranes as the centers of the circles and selected the 20 km as the radius of each circle to create buffers. Finally extracted the NL value of all unduplicated occurrences of all species of five functional groups located within buffers and repeated this 1,000 times. For each time, the number of selected occurrences was the same as the number of occurrences of four species of cranes. We then calculated the SES for crane species. These steps were followed to calculate the SES for other groups also. NL values were calculated in ArcGIS 10.5, and the bootstrapping procedure in Matlab 2016a.

#### 2.2.3 Relationship between standardized effect size (SES) and threat status

SES was used to represent relative habitat association. The IUCN threat status categories CR, EN, and VU were considered threatened (threat) and those ranked NT and LC as unthreatened (nonthreat) species (Hu et al., 2017). We considered range size (RS) of each waterbird species as mediator because it accurately predicts threat status (TS) of waterbirds (IUCN, 2016; Ramesh, Gopalakrishnac, Barved, & Melnick, 2017), and we expect to know how SES is related to TS once RS is accounted for. Range sizes were based on the distribution range of species in China as defined by BirdLife International (http://datahzone.birdlife.org/home). Also, species are not phylogenetically independent, and threat status or SES may vary across the different functional groups we analyze, which could generate spurious associations (or lack of association) between SES and TS. We consider functional group (FG) to be a random effect in analysis.

We first test whether the relationship between SES and RS is significant and then determine whether RS can accurately predict TS. We consider that SES can predict threat status if SES can accurately predict RS, and RS can accurately predict TS. Specific analytical steps are as follows:

1. We first used an ordinary least squares (OLS) in Stata 15 to test whether SES can accurately predict RS. We considered FG to be a random variable (Equation 4)

(4) $$\ln\text{RS}=\alpha +\beta_{1}\text{SES}+\text{FG}+\varepsilon$$

*α* is a constant term, *β*_1_ is a regression coefficient of SES, and *ε* is a stochastic error term.

- We then use a generalized linear model (GLM) in Stata 15 to test whether RS can accurately predict TS. We consider FG to be a random variable (Equation 5). The threat status of a threatened species was assigned a value of 1 and an unthreatened species a value of 0.

(5) $$\text{Logit}\left(\text{TS}\right)=\alpha +\beta_{1}\ln\text{RS}+\text{FG}+\varepsilon$$

*α* is a constant term, *β*_1_ is a regression coefficient of RS, and *ε* is a stochastic error term.

- We used the Sobel–Goodman mediation tests (Sobel) in Stata 15 to check whether the mediating effect of RS is significant for influencing the relationship between SES and TS.

#### 2.2.4 Analysis of protection status

All species were divided into one of two categories: (a) those significantly associated with natural landscapes and (b), those significantly associated with human‐dominated landscapes or without any significant land association. We then assigned the protection status of each species in five functional groups following the National Protected Species (NPS) list of Key Protected Wildlife, of the *Wildlife Protection Law of China* (Ministry of Forestry in the People's Republic of China, 1988), including NPS Classes I and II (a checklist is presented in Appendix S2); species in NPS Class I or II were assigned as protected and others as not protected (Hu et al., 2017). Finally, we used a Mann–Whitney *U* test in SPSS 22.0 to compare for differences in mean number of species protected in five functional groups of two categories.

## 3 RESULTS

### 3.1 Bootstrapping procedure

**FIGURE 3:**
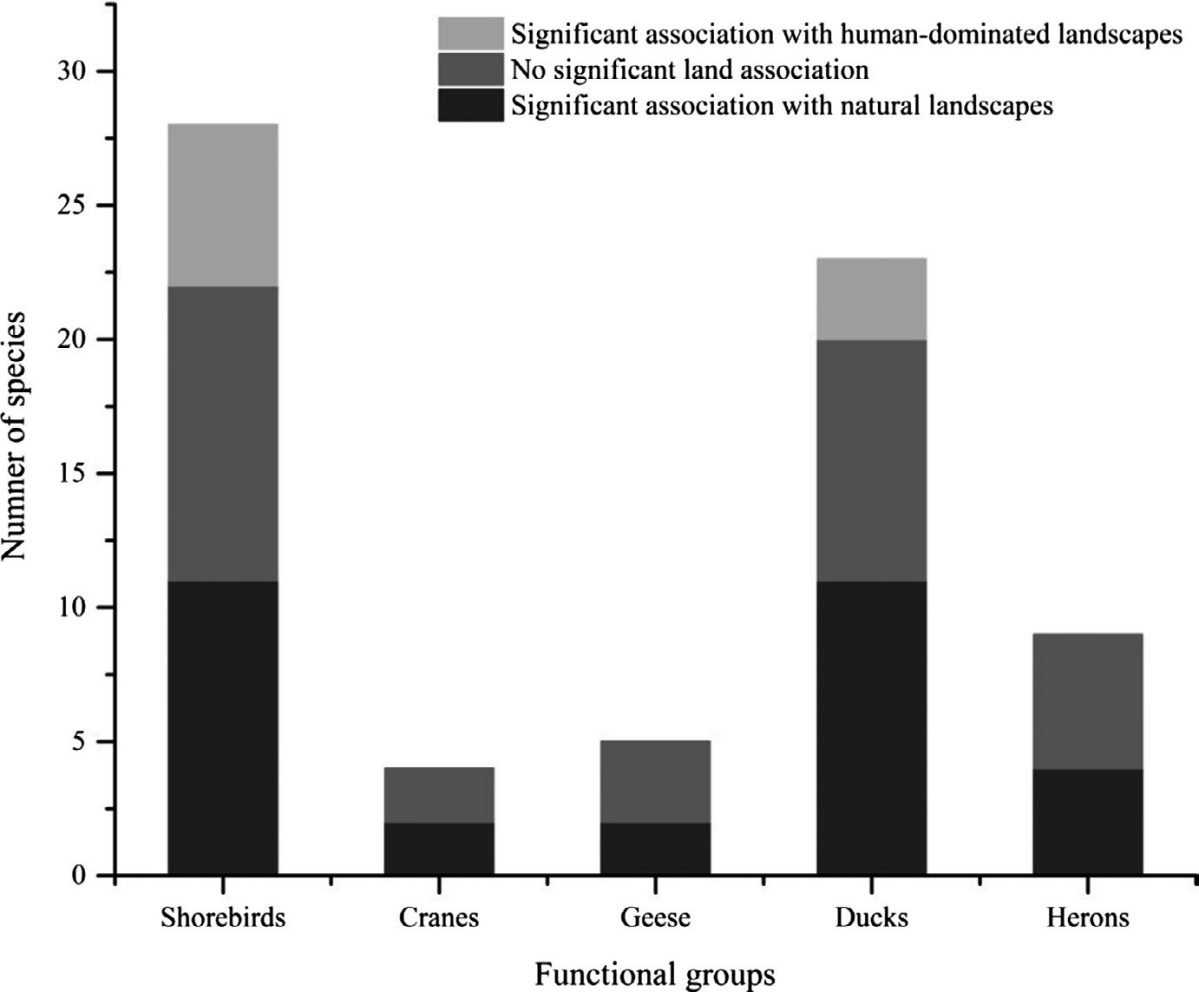
Numbers of species in different functional groups significantly associated with human‐dominated landscapes, no significant land association, or significantly associated with natural landscapes

Of the 69 waterbird species analyzed, 30 (43.5%) were significantly associated with natural landscapes, including 11 shorebird species, 2 crane species, 2 geese species, 11 duck species, and 4 heron species. A further 9 (13.0%) species were significantly associated with human‐dominated landscapes, including 6 shorebird species and 3 duck species. The remaining 30 (43.5%) species were not significantly associated with landscape types, comprising 11 shorebird species, 2 crane species, 3 geese species, 9 duck species, and 5 heron species (Figure 3, Appendix S2). SES values for the functional groups of geese (7.20), cranes (5.32), and ducks (4.12) were higher than those of shorebirds (1.99) and herons (0.18), indicating that geese and cranes are more dependent on natural landscapes than are other functional groups.

### 3.2 Relationship between standardized effect size (SES) and threat status

Results for OLS revealed the relationship between SES and range size (RS) to be significant (*N* = 69, *β*_1_ = −318.05, *t *= −3.28, *p* = .002); the higher SES, the lower the RS. GLM results indicated the relationship between RS and threat status to be significant (*N* = 61, *β*_1_ = −1.66, *t* = 3.19, *p* = .001); the lower the RS, the higher the threat status. Sobel results indicate RS significantly mediates the relationship between SES and threat status (*Z* = 2.498, *p* = .012). We conclude that the relationship between SES and threat status was significant when the factor “range size” is considered as the mediator, and that the higher the SES, the higher the threat status.

### 3.3 Analysis of protection status

**FIGURE 4:**
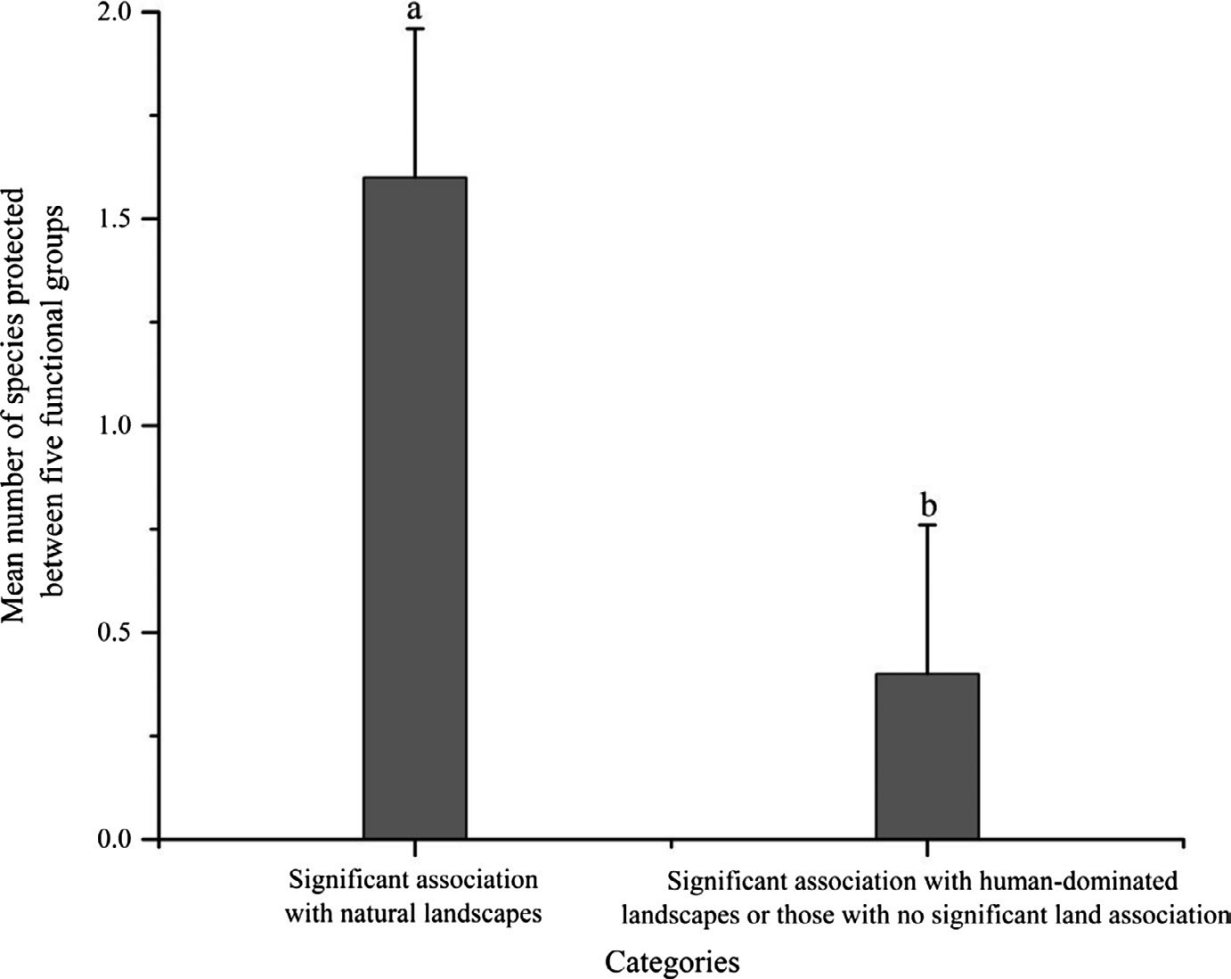
Mean number of species protected between five functional groups in categories significantly associated with natural landscapes and significantly associated with human‐dominated landscapes or lacking significant land association. Letters (a, b) above standard error bars indicate a significant difference between categories

Of species significantly associated with natural landscapes, 26.7% were afforded NPS protection, compared to only 3.33% of species that were significantly associated with human‐dominated landscapes or were not significantly associated with any particular land type. The difference between the two categories was significant (*Z *= −1.972, *p* = .049) (Figure 4; Appendix S2).

## 4 DISCUSSION

Identifying the sensitivity of waterbirds to landscape in China can reduce unnecessary effort to protect species that are more dependent on human‐dominated landscapes. Of our 69 species, 43.5% were significantly associated with natural landscapes. The higher the association was with natural landscapes, the higher the threat status of a species when considering range size as the mediator. This implies that loss of associated habitat will increase risks for these species, compared to others (Dolman & Sutherland, 2010; Galbraith et al., 2002). Many of these species (*Numenius madagascariensis*, *Charadrius mongolus*, *Xenus cinereus*, *Grus vipio*) are highly dependent on natural landscapes, which have declined dramatically in China since 1960 (Barter, 2002; Burger, Niles, & Clark, 1997; Chen, Yang, & Lu, 2015; Li et al., 2019). The populations of these species have all declined in recent years, due largely to habitat loss (Studds et al., 2017; Wang, Fraser, & Chen, 2017).

Associations with natural landscapes were higher for cranes, geese, and ducks than for shorebirds and herons. To some extent, this suggested that shorebirds and herons were less sensitive of natural habitat loss than other functional groups. In recent decades, the conversion of tidal flats in China has forced the majority of shorebirds to use alternative habitats (Basso, Fonseca, Drever, & Navedo, 2017; Jackson et al., 2019). A growing number of studies have also demonstrated shorebirds now exploit artificial fish ponds and saltpans for stopovers during their migration (Jackson et al., 2019; Sripanomyom, Round, Savini, Trisurat, & Gale, 2011; Yasué & Dearden, 2009). Some large shorebird species even prefer to feed in artificial habitats (Lei et al., 2018; Yasué & Dearden, 2009).

Species significantly associated with natural landscapes were eight times more likely to be legally protected or regarded as of conservation concern by wildlife protection law than species significantly associated with human‐dominated landscapes or species with no significant land association. However, 73.3% of species significantly associated with natural landscapes were not listed in existing wildlife protection law, such as the globally threatened *Calidris pygmaea* and *N. madagascariensis*. We suggested that governmental conservation agencies should pay more attention to the species associated with natural landscapes, to prevent further decline in their populations and habitats.

The method of bootstrapping procedures only reveals the relative sensitivity of species to landscape. This does not mean that the less sensitive species are absolutely insensitive to landscapes changes, but that some are more likely to occur in human‐dominated environments than ecologically similar species. For example, the ducks *Melanitta fusca*, *Mergellus albellus,* and *Anas poecilorhyncha* were significantly associated with human‐dominated landscapes, while *Mergus squamatus* and *Aythya ferina* were significantly associated with natural landscapes. Even though the first three of these species are often found in artificial lakes and reservoirs (Kloskowski, Green, Polak, Bustamante, & Krogulec, 2010), this did not mean them insensitive to natural landscapes loss, but it does imply that the latter two species are more sensitive to natural habitat loss. In addition, bootstrapping procedures can only conduct this analysis within group of similar habit due to our assumption was that species within in a group of similar habits would have a similar likelihood of being reported.

## 5 CONCLUSIONS

Among 69 waterbird species studied in China, 30 sensitive species were significantly associated with natural landscapes, and associations with natural landscapes were higher for cranes, geese, and ducks than for shorebirds and herons. The higher the association was with natural landscapes, the higher the threat status of a species when considering range size as the mediator. Sensitive species significantly associated with natural landscapes can acquire more protection than less sensitive species significantly associated with human‐dominated landscapes. We suggest taxonomic targets for conservation, particularly species that are more dependent on natural landscapes than others. We also suggest that more citizen science data need to be collected with improvement of standardization and protocol, so as to benefit to conservation and management of waterbirds and their habitats with higher scientific value of these data.

## CONFLICT OF INTEREST

None declared.

## AUTHOR CONTRIBUTIONS

**Houlang Duan:** Data curation (lead); Formal analysis (lead); Investigation (lead); Methodology (lead); Software (lead); Supervision (lead); Writing‐original draft (lead); Writing‐review & editing (lead). **Shaoxia Xia:** Writing‐review & editing (supporting). **Xiubo Yu:** Conceptualization (lead); Supervision (lead); Writing‐review & editing (supporting). **Yu Liu:** Supervision (supporting). **Jiakun Teng:** Software (supporting). **Yuehan Dou:** Writing‐review & editing (supporting).

## Supporting information

Appendix S1‐S2

## Data Availability

The records of species supporting this study can be acquired in the websites of eBird in the USA (https://ebird.org/home), the Global Biodiversity Information Network (GBIF) (http://www.gbifchina.org/), and from BirdReport in China (http://www.birdreport.cn/). The land use and land cover data are available from the Data Center for Resources and Environmental Sciences at the Chinese Academy of Sciences (RESDC) (http://www.resdc.cn).
