# Supplementary material for: Using citizen science data to inform the relative sensitivity of waterbirds to natural versus human‐dominated landscapes in China
Source: Ecol Evol. 2020 Jun 9;10(14):7233–41. doi: 10.1002/ece3.6449 (PMC7391315; doi:10.1002/ece3.6449)
Supplement: Supplementary file 1 — Appendix S1‐S2 [file ECE3-10-7233-s001.docx]

**Appendix 1.** Waterbird summary statistic data. IUCN status: critically endangered (CR), endangered (EN), vulnerable (VU), near threatened (NT), least concern (LC).

| Functional group | Code | English name | Latin name | Occurrence numbers | IUCN Red list category |
| --- | --- | --- | --- | --- | --- |
| Shorebirds | 1 | Pintail Snipe | *Gallinago stenura* | 94 | LC |
|  | 2 | Common Snipe | *Gallinago gallinago* | 975 | LC |
|  | 3 | Black-tailed Godwit | *Limosa limosa* | 375 | NT |
|  | 4 | Bar-tailed Godwit | *Limosa lapponica* | 191 | NT |
|  | 5 | Eastern Curlew | *Numenius madagascariensis* | 172 | EN |
|  | 6 | Marsh Sandpiper | *Tringa stagnatilis* | 478 | LC |
|  | 7 | Common Greenshank | *Tringa nebularia* | 1034 | LC |
|  | 8 | Nordmann's Greenshank | *Tringa guttifer* | 39 | EN |
|  | 9 | Green Sandpiper | *Tringa ochropus* | 1443 | LC |
|  | 10 | Terek Sandpiper | *Xenus cinereus* | 216 | LC |
|  | 11 | Common Sandpiper | *Actitis hypoleucos* | 1629 | LC |
|  | 12 | Asian Dowitcher | *Limnodromus semipalmatus* | 71 | NT |
|  | 13 | Red Knot | *Calidris canutus* | 113 | NT |
|  | 14 | Sanderling | *Calidris alba* | 136 | LC |
|  | 15 | Spoon-billed Sandpiper | *Calidris pygmaea* | 64 | CR |
|  | 16 | Sharp-tailed Sandpiper | *Calidris acuminata* | 189 | LC |
|  | 17 | Dunlin | *Calidris alpina* | 433 | LC |
|  | 18 | Curlew Sandpiper | *Calidris ferruginea* | 241 | NT |
|  | 19 | Eurasian Oystercatcher | *Haematopus ostralegus* | 139 | NT |
|  | 20 | Black-winged Stilt | *Himantopus himantopus* | 914 | LC |
|  | 21 | Pied Avocet | *Recurvirostra avosetta* | 562 | LC |
|  | 22 | Kentish Plover | *Charadrius alexandrinus* | 911 | LC |
|  | 23 | Lesser Sand Plover | *Charadrius mongolus* | 135 | LC |
|  | 24 | Eurasian Curlew | *Numenius arquata* | 430 | NT |
|  | 25 | Grey Plover | *Pluvialis squatarola* | 137 | LC |
|  | 26 | Little Ringed Plover | *Charadrius dubius* | 1276 | LC |
|  | 27 | Wood Sandpiper | *Tringa glareola* | 799 | LC |
|  | 28 | Greater Sand Plover | *Charadrius leschenaultii* | 238 | LC |
| Cranes | 29 | Siberian Crane | *Grus leucogeranus* | 186 | CR |
|  | 30 | White-naped Crane | *Grus vipio* | 76 | VU |
|  | 31 | Hooded Crane | *Grus monacha* | 81 | VU |
|  | 32 | Red-crowned Crane | *Grus japonensis* | 54 | EN |
| Geese | 33 | Swan Goose | *Anser cygnoides* | 164 | VU |
|  | 34 | Bean Goose | *Anser fabalis* | 94 | LC |
|  | 35 | Greater White-fronted Goose | *Anser albifrons* | 152 | LC |
|  | 36 | Greylag Goose | *Anser anser* | 184 | LC |
|  | 37 | Bar-headed Goose | *Anser indicus* | 171 | LC |
| Ducks | 38 | Ruddy Shelduck | *Tadorna ferruginea* | 677 | LC |
|  | 39 | Common Shelduck | *Tadorna tadorna* | 241 | LC |
|  | 40 | Gadwall | *Anas strepera* | 468 | LC |
|  | 41 | Falcated Duck | *Anas falcata* | 204 | NT |
|  | 42 | Eurasian Wigeon | *Anas penelope* | 343 | LC |
|  | 43 | Mallard | *Anas platyrhynchos* | 980 | LC |
|  | 44 | Spot-billed Duck | *Anas poecilorhyncha* | 1045 | LC |
|  | 45 | Northern Shoveler | *Anas clypeata* | 317 | LC |
|  | 46 | Northern Pintail | *Anas acuta* | 227 | LC |
|  | 47 | Garganey | *Anas querquedula* | 172 | LC |
|  | 48 | Eurasian Teal | *Anas crecca* | 874 | LC |
|  | 49 | Red-crested Pochard | *Netta rufina* | 295 | LC |
|  | 50 | Ferruginous Pochard | *Aythya nyroca* | 205 | NT |
|  | 51 | Baer's Pochard | *Aythya baeri* | 40 | CR |
|  | 52 | Tufted Duck | *Aythya fuligula* | 234 | LC |
|  | 53 | Greater Scaup | *Aythya marila* | 43 | LC |
|  | 54 | White-winged Scoter | *Melanitta fusca* | 53 | VU |
|  | 55 | Common Goldeneye | *Bucephala clangula* | 343 | LC |
|  | 56 | Smew | *Mergellus albellus* | 486 | LC |
|  | 57 | Scaly-sided Merganser | *Mergus squamatus* | 140 | EN |
|  | 58 | Common Merganser | *Mergus merganser* | 925 | LC |
|  | 59 | Common Pochard | *Aythya ferina* | 499 | VU |
|  | 60 | Red-breasted Merganser | *Mergus serrator* | 118 | LC |
| Herons | 61 | Grey Heron | *Ardea cinerea* | 2097 | LC |
|  | 62 | Purple Heron | *Ardea purpurea* | 393 | LC |
|  | 63 | Great Egret | *Casmerodius albus* | 1603 | LC |
|  | 64 | Intermediate Egret | *Ardea intermedia* | 662 | LC |
|  | 65 | Cattle Egret | *Bubulcus ibis* | 1153 | LC |
|  | 66 | Chinese Pond Heron | *Ardeola bacchus* | 113 | LC |
|  | 67 | Eurasian Spoonbill | *Platalea leucorodia* | 438 | LC |
|  | 68 | Black-faced Spoonbill | *Platalea minor* | 149 | EN |
|  | 69 | Chinese Egret | *Egretta eulophotes* | 358 | VU |

**Appendix 2.** Standardized effect size (SES), mean natural landscape value, protection status and range size for waterbird species.

| Functional groups | Species name | Standardized effect size | Mean natural landscape value | Protection status | Range size(1000×m^2^) |
| --- | --- | --- | --- | --- | --- |
| Shorebirds | *Charadrius mongolus^a^* | 5.454 | 0.576 | Not listed | 822.347 |
|  | *Calidris pygmaea^a^* | 3.315 | 0.509 | Not listed | 37.427 |
|  | *Recurvirostra avosetta^a^* | 3.096 | 0.480 | Not listed | 1631.690 |
|  | *Calidris ferruginea^a^* | 2.550 | 0.486 | Not listed | 1340.712 |
|  | *Tringa guttifer^a^* | 2.545 | 0.501 | Class II | 47.672 |
|  | *Pluvialis squatarola^a^* | 2.534 | 0.505 | Not listed | 708.018 |
|  | *Limnodromus semipalmatus^a^* | 2.225 | 0.491 | Not listed | 362.683 |
|  | *Limosa lapponica^a^* | 2.154 | 0.462 | Not listed | 597.701 |
|  | *Numenius arquata^a^* | 2.150 | 0.476 | Not listed | 4708.106 |
|  | *Xenus cinereus^a^* | 2.133 | 0.474 | Not listed | 1925.799 |
|  | *Numenius madagascariensis^a^* | 2.028 | 0.473 | Not listed | 830.181 |
|  | *Limosa limosa* | 1.683 | 0.463 | Not listed | 5860.115 |
|  | *Calidris canutus* | 1.424 | 0.461 | Not listed | 1013.91 |
|  | *Tringa ochropus* | 1.320 | 0.419 | Not listed | 4770.612 |
|  | *Calidris alba* | 0.341 | 0.444 | Not listed | 656.027 |
|  | *Haematopus ostralegus* | 0.307 | 0.440 | Not listed | 1492.981 |
|  | *Himantopus himantopus* | 0.202 | 0.442 | Not listed | 4654.866 |
|  | *Calidris acuminata* | -0.629 | 0.381 | Not listed | 809.753 |
|  | *Charadrius alexandrines* | -1.046 | 0.432 | Not listed | 2238.130 |
|  | *Charadrius leschenaultii* | -1.086 | 0.393 | Not listed | 911.428 |
|  | *Gallinago stenura* | -1.196 | 0.370 | Not listed | 2056.338 |
|  | *Calidris alpina* | -1.432 | 0.409 | Not listed | 477.547 |
|  | *Tringa stagnatilis^b^* | -2.651 | 0.398 | Not listed | 3151.873 |
|  | *Tringa nebularia^b^* | -2.992 | 0.415 | Not listed | 4875.464 |
|  | *Tringa glareola^b^* | -3.319 | 0.407 | Not listed | 5258.342 |
|  | *Gallinago gallinago^b^* | -3.688 | 0.406 | Not listed | 5313.043 |
|  | *Charadrius dubius^b^* | -3.831 | 0.413 | Not listed | 5051.354 |
|  | *Actitis hypoleucos^b^* | -4.777 | 0.412 | Not listed | 6710.601 |
| Cranes | *Grus leucogeranus^a^* | 3.886 | 0.683 | Class I | 3.685 |
|  | *Grus vipio^a^* | 2.258 | 0.727 | Class II | 240.527 |
|  | *Grus japonensis* | 0.158 | 0.645 | Class I | 245.828 |
|  | *Grus monacha* | -0.499 | 0.643 | Class I | 236.673 |
| Geese | *Anser anser^a^* | 3.514 | 0.743 | Class II | 2244.503 |
|  | *Anser albifrons^a^* | 3.141 | 0.618 | Not listed | 310.275 |
|  | *Anser fabalis* | 1.682 | 0.637 | Not listed | 2872.826 |
|  | *Anser indicus* | 1.070 | 0.714 | Not listed | 434.75 |
|  | *Anser cygnoides* | -1.050 | 0.585 | Not listed | 808.786 |
| Ducks | *Tadorna ferruginea^a^* | 9.223 | 0.623 | Not listed | 2312.096 |
|  | *Netta rufina^a^* | 9.049 | 0.731 | Not listed | 950.301 |
|  | *Aythya ferina^a^* | 4.562 | 0.583 | Not listed | 3165.189 |
|  | *Anas strepera^a^* | 4.486 | 0.580 | Not listed | 1452.324 |
|  | *Anas penelope^a^* | 4.202 | 0.590 | Not listed | 3883.363 |
|  | *Tadorna tadorna^a^* | 3.337 | 0.589 | Not listed | 1261.690 |
|  | *Anas clypeata^a^* | 2.962 | 0.570 | Not listed | 4027.785 |
|  | *Aythya nyroca^a^* | 2.941 | 0.589 | Not listed | 3495.607 |
|  | *Anas acuta^a^* | 2.613 | 0.574 | Not listed | 4574.152 |
|  | *Aythya fuligula^a^* | 2.466 | 0.558 | Not listed | 4106.033 |
|  | *Mergus squamatus^a^* | 2.138 | 0.518 | Class I | 225.009 |
|  | *Anas falcate* | 1.266 | 0.511 | Not listed | 1207.858 |
|  | *Aythya baeri* | 1.111 | 0.461 | Not listed | 3405.071 |
|  | *Bucephala clangula* | 0.895 | 0.523 | Not listed | 3105.031 |
|  | *Anas querquedula* | 0.444 | 0.523 | Not listed | 3760.861 |
|  | *Mergus merganser* | 0.163 | 0.517 | Not listed | 4499.081 |
|  | *Anas platyrhynchos* | -0.441 | 0.511 | Not listed | 5929.860 |
|  | *Aythya marila* | -0.931 | 0.399 | Not listed | 4895.540 |
|  | *Mergus serrator* | -1.307 | 0.416 | Not listed | 4386.825 |
|  | *Anas crecca* | -1.320 | 0.502 | Not listed | 4953.384 |
|  | *Anas poecilorhyncha^b^* | -2.154 | 0.487 | Not listed | 4102.356 |
|  | *Melanitta fusca^b^* | -2.949 | 0.312 | Not listed | 3246.675 |
|  | *Mergellus albellus^b^* | -3.626 | 0.455 | Not listed | 2190.462 |
| Herons | *Bubulcus ibis^a^* | 3.033 | 0.461 | Not listed | 4701.065 |
|  | *Platalea leucorodia^a^* | 2.905 | 0.484 | Class II | 820.880 |
|  | *Platalea minor^a^* | 2.171 | 0.487 | Class II | 19.398 |
|  | *Egretta eulophotes^a^* | 1.987 | 0.471 | Class II | 40.705 |
|  | *Ardeola bacchus* | 1.762 | 0.498 | Not listed | 733.484 |
|  | *Casmerodius albus* | 1.450 | 0.457 | Not listed | 6824.973 |
|  | *Ardea purpurea* | 0.116 | 0.434 | Not listed | 3133.324 |
|  | *Ardea intermedia* | -1.323 | 0.427 | Not listed | 6123.342 |
|  | *Ardea cinerea* | -1.428 | 0.440 | Not listed | 5274.857 |

Superscripts indicate a significant association with ^‘a’^ natural landscapes, and ^‘b’^ human-dominated landscapes. Positive SES values indicate a species occurred in more natural landscapes than the output from the mean natural landscape value of 1000 random samples. Negative SES values indicate a species occurs in more human-dominated landscapes than the output from the mean natural landscape value of 1000 random samples. Species in NPS Class I or II were assigned as protected, and others ‘Not listed’ as not protected.
